# The Concentrations of Fatty Acids, Cholesterol and Vitamin E in Cooked Longissimus, Semitendinosus, Psoas Major and Supraspinatus Muscles from Cattle Offered Grass Only, Concentrates Ad Libitum or Grass Silage Supplemented with Concentrates

**DOI:** 10.3390/foods14050747

**Published:** 2025-02-22

**Authors:** Aidan P. Moloney, Cormac McElhinney, Raquel Cama-Moncunill, Edward G. O′Riordan, Frank J. Monahan

**Affiliations:** 1 Teagasc, Animal & Grassland Research and Innovation Centre, Grange, Dunsany, Co., C15 PW93 Meath, Ireland; 2 School of Agriculture and Food Science, University College Dublin, Belfield, D04 C1P1 Dublin 4, Ireland

**Keywords:** beef, grass, muscle, CLA, omega-3 fatty acids

## Abstract

There is growing interest among consumers in the nutritional value of the food they consume. The objectives of this study were (1) to document the nutritional value, with a focus on the fatty acid profile, of beef from cattle from one commercial production system that only ever received grass-based feed ingredients compared with similar animals finished in more conventional, i.e., with high-concentrate or concentrate-supplemented grass silage, production systems; (2) to determine the effect of the dietary treatments on muscles representing a range in intramuscular fat concentrations and commercial value, viz *supraspinatus*, *semitendinosus*, *longissimus lumborum* and *psoas major*; and (3) to determine if the fatty acid profile can be used to distinguish between different beef muscles. Dietary treatment and muscle type influenced the concentration of omega-3 polyunsaturated fatty acids and conjugated linoleic acid, with the highest concentrations observed in *psoas major* from cattle fed grass only. These data should be of use to the meat industry and to those updating nutritional databases. The possibility of discriminating beef according to its dietary background based on the fatty acid profile was confirmed. While this approach was moderately successful with respect to the separation of *supraspinatus*, *longissimus lumborum* and *psoas major*, discrimination between the more valuable *longissimus lumborum* and the lower-value *semitendinosus* is more challenging.

## 1. Introduction

Interest among consumers in the nutritional value of the food they consume has resulted in a growing preference for foods that are perceived as being healthier [1]. In general, beef from grazed, grass-fed animals has a higher vitamin E concentration and a better profile of fatty acids from a human nutrition perspective compared to beef from alternative production systems [2]. This has contributed to the growing interest in beef from animals finished off pasture [3]. Despite the large literature on this topic, many publications on grass-fed beef describe studies where animals were finished at pasture for some relatively short period prior to slaughter although their pre-finishing diet was based on concentrates or not reported. Very few studies, however, report the fatty acid profile or nutritional value of beef from cattle that were fed grazed or conserved pasture throughout their entire life, i.e., truly ”grass-fed”. The first objective of this study was to document the nutritional value, with a focus on the fatty acid profile, of beef from cattle from one particular commercial production system that had never received non-grass-based feed ingredients in comparison with similar animals finished in more conventional, i.e., with high-concentrate or concentrate-supplemented grass silage, production systems. An ancillary objective was to determine the impact of supplementation of the concentrate in the latter production system with a ruminally-protected source of linolenic acid to mimic the post-ruminal supply of this fatty acid in the grazing cattle.

The majority of studies on the influence of diet on the fatty acid composition of beef only consider the longissimus muscle (LM) as an “indicator” muscle. There is a range of muscles in the beef carcass that differ in composition and commercial value [4]. The second objective of this study was to determine the effect of the dietary treatments on muscles that currently have a higher or lower value than the LM and that represent a range of intramuscular fat (IMF) concentrations.

An ability to authenticate a food is critical in preventing fraudulent claims or practices, as well as in securing and maintaining consumer confidence in a product. The potential of using the fatty acid profile to distinguish pasture-fed beef from beef produced under other dietary regimens has been demonstrated [5,6]. Given the range in value of different cuts/muscles within the beef carcass, methods to authenticate high-value or premium muscles would also benefit the beef industry. The third objective of this study was to determine if the fatty acid profile could also be used to distinguish between different beef muscles.

## 2. Materials and Methods

### 2.1. Animal Management and Sample Collection

This study was conducted at Teagasc, Grange Research Centre, Ireland (longitude, 6°40′ W; latitude, 53°30′ N; elevation, 92 m above sea level) and was briefly described in [7]. All animal procedures were conducted under an experimental license (AE 19132/P034) issued by the Health Products Regulatory Body in accordance with the Irish Medicines Board Acts 1995 and 2006 (authorisation of a project pursuant to part 5 of European Union Regulations 2012 (S.I. No. 543 of 2012)) and approved by the Teagasc Animal Ethics Committee (TAEC license number: 61/2014, 19 September 2014). Sixty spring-born, Angus-sired (Angus-sired dams) heifers were purchased at approximately 8 months of age from individual farms after verification that they had never received concentrates to date. They were acclimatised to slatted floor accommodation and offered grass silage ad libitum for 1 month. The animals were weighed, blocked according to bodyweight (BW) (mean initial BW, 275 kg; SD = 27 kg), balanced for birth date and within-block, assigned at random to one of four dietary treatments. Within treatment, animals were assigned at random to pens (13 m^2^) of five animals in a concrete, slatted-floor shed. One treatment group was offered standard concentrates (Table 1) ad libitum and 5 kg medium-digestibility grass silage (Table 1) per animal daily until slaughter on 8 June (CONC), day 188 of the experiment. Two groups were offered the medium-digestibility grass silage ad libitum and 2 kg of standard concentrates (Table 1) (GSS and GSL). The fourth treatment group was offered high-digestibility grass silage (Table 1) ad libitum plus 53 g mineral supplement daily. The latter three groups were turned out to pasture on 4 April (day 127 of the experiment) and rotationally grazed in groups of 5 animals per treatment in a predominantly Lolium perenne L. pasture. Daily grass allowance was based on a pre-grazing herbage mass of 2000 kg dry matter (DM)/ha estimated using a rising plate meter (Filips Folding Plate Meter, Jenquip, Feilding, New Zealand), with an individual target of 25 g DM/kg BW. This was achieved by varying the size of the grazing area, and animals were offered a fresh allowance every 2 to 3 days, without access to the previous grazing area. One of the groups previously offered grass silage and concentrates was housed on 10 July (day 230 of the experiment) and offered finishing grass silage (Table 1) ad libitum and 3.2 kg of the standard concentrate until slaughter on 21 September (GSS), day 293 of the experiment. The other group previously offered grass silage and concentrates was also housed on 10 July and offered finishing grass silage ad libitum and 3.2 kg of a concentrate fortified with a mixture of linseed oil and ruminally-protected linseeds (Protected linseed, Troew Nutrition, Belfast, Northern Ireland) (Table 1) until slaughter on 22 September (GSL), day 294 of the experiment. Both groups were housed in pens of five animals that grazed as a group while at pasture. The group previously offered the high-digestibility grass silage only remained at pasture until slaughter on 11 November (GRASS), day 336 of the experiment. All animals were weighed periodically and were slaughtered at a commercial abattoir when they reached a group mean BW to yield a mean target carcass weight of 260 kg. For all groups, daily concentrate and silage intake was recorded when the animals were housed. The standard concentrate was manufactured by Lakeland Dairies Co-Op Society Ltd., Cavan, Ireland. The GSL concentrate was manufactured periodically on site using a cement mixer. Thus, 15 kg linseed oil and 49 kg ruminally-protected linseeds (Trouw Nutrition, Belfast, Ireland) were added to 1000 kg of the standard concentrate and mixed for 1 h. The concentrate was stored in a dry container and hand-mixed before feeding.

Silage samples collected daily, concentrates samples collected twice per week and grass samples collected each time animals were moved were stored frozen at −20 °C. Silage and grass samples were placed in a freezer within 5 min of collection and grass samples within 20 min.

Animals were slaughtered at Kepak Ltd., Clonee, County Meath, Ireland. All slaughter and dressing procedures complied with Regulations (EC) No. 1099/2009 and No. 853/2004, and electrical stimulation was not applied. Post slaughter, carcasses were weighed, graded for conformation and fatness (Table 2) and placed in a chill. Forty-eight h post mortem, the right *Longissimus* muscle (LM) from the 10th rib to the posterior end (3 rib striploin commercial cut) and the intact *semitendinosus* (ST), *psoas major* (PM) and *supraspinatus* (SUP) muscles were excised from each carcass. The muscles were vacuum-packaged and transferred to the Teagasc Food Research Centre, Ashtown, Dublin 15, and stored at 2 °C for a further 12 days. On day 14 post mortem, the subcutaneous fat was removed from the LM and sub-sampled. All muscles were then cut into steaks 2.5 cm in thickness, placed in bags pre-labelled for subsequent analysis, vacuum-packaged (as were the fat samples) and stored at −20 °C until required.

### 2.2. Cooking Protocol

Muscle samples were allowed to thaw in a fridge. Once thawed, non-muscular visible fat (subcutaneous and/or intermuscular) was removed using a sharp knife, and samples were vacuum-packed. Samples were cooked in a water bath (Memmert ™ GmbH, Schwabach, Germany) that was set to 72 °C. Samples kept in the water bath until the core temperature of the sample reached 72 °C, measured using a calibrated temperature probe (HI 904, Hanna Foodcare Instruments, Bedfordshire, UK). Once cooked, samples were cooled in ambient conditions, and the juice of the cooked steak was drained before the meat sample was homogenised using a Robot Coupe R301 Ultra food processor (Robot Coupe S.N.C., Vincennes, France) for 30 s. Homogenised samples were then subjected to chemical analysis as described below. A vacuum-packaged sample of fat from each animal, pooled according to treatment, was cooked similarly but without any liquid removal.

### 2.3. Chemical Analysis: Tissue

#### 2.3.1. Moisture and Protein

Homogenised samples were freeze-dried (Edwards Supermodulyo F056, pressure 0.02 atm, condenser temperature −60 °C, Fisher Scientific, Dublin, Ireland), and the moisture concentration was calculated as the loss in weight. Protein in the freeze-dried residue was determined using a LECO protein analyser based on the Dumas method (Model FP-428, Leco Corporation, St. Joseph, MI, USA) [8].

#### 2.3.2. Fatty Acids

All reagents were purchased from Sigma.

Aldrich (Sigma Aldrich Ireland Ltd, Arklow, Ireland). Fatty acids were extracted and methylated in duplicate using the rapid microwave-assisted method described by Brunton [9]. Thus, homogenised, cooked beef was accurately weighed (1 g) in a MARS™ Xpress extraction vessel. Internal standard (C23:0, 0.1% in hexane) was added to each sample, after which a magnetic stirring bar was added to the tube. For saponification, 10 mL of 2.5% KOH in methanol was added to each vessel. Samples were placed in the MARS™ 6 microwave for 8 min (4 min to reach 130 °C and 4 min held at 130 °C), continuously stirred, then allowed to cool. For esterification, 15 mL of methanolic acetyl chloride was then added to the vessel. Samples were placed in the MARS™ 6 microwave for 6 min (4 min to reach 120 °C and 2 min held at 120 °C), continuously stirred, then allowed to cool. Pentane (GCMS grade, 10 mL) was added to the vessel, and the vessel was sealed and inverted gently 10 times by hand. Then, 20 mL of saturated NaCl solution was added to each vessel, and again, the vessel was inverted gently 10 times. Vessels remained unopened and upright for 30 min at room temperature to facilitate phase separation. Then, 2 mL of the top layer (pentane) in the vessel was transferred using a plastic Pasteur pipette into a GC vial containing 0.2 g anhydrous sodium sulphate (Na_2_SO_4_) (drying agent). Fatty acid methylesters (FAMEs) were analysed using a Varian 3500 GLC (Varian, Harbor City, CA, USA) and a 100 m CP-Sil 88 column (100 m × 0.25 mm i.d., 0.2 µm film thickness, Supelco, Bellefonte, PA, USA). Hydrogen was the carrier gas, and GC conditions were as described previously [10].

#### 2.3.3. Cholesterol and Vitamin E

An internal standard (50 µL of 50 mg/mL dichloromethanolic 5-Cholestan-3β-ol) was added to a centrifuge tube, which was placed in a sample concentrator set at 40 °C with nitrogen stream for 10 min; then, 0.5 g of cooked meat was added to the tube. Saponification and derivatisation were carried out as described by Grasso et al. [11]. Cholesterol was identified using an Agilent 7890B GC-FID and the column conditions reported in [11] and quantified relative to the internal standard using Agilent software (Open lab Chemstation). Vitamin E was measured as previously described [12].

#### 2.3.4. Vitamin A and Beta-Carotene

The concentrations of vitamin A and beta-carotene in muscle and fat were measured in a commercial laboratory (Advanced Laboratory Testing Ltd. (ALT), Newbridge, County Kildare, Ireland). Homogenised samples were saponified with alcoholic KOH, and the retinol and carotenes were extracted into hexane, which was then evaporated, after which the residue was re-dissolved in ethanol. The concentration of retinol was determined using reverse-phase HPLC with UV detection (BS/EN 128-1:2000: Determination of Vitamin A by HPLC. Part 1: Measurement of all-trans = retinol and 13-cis-retinol (limit of detection, 10 μg/100 g; relative uncertainty = 8.6%)). The concentration of alpha- and ß-carotene was determined using reverse-phase HPLC with visible detection (BS/EN 12823-1:2000: Determination of Vitamin A by HPLC. Part 1: Measurement of alpha- and beta-carotenes (limit of detection, 10 μg/100 g; relative uncertainty = 8.9%)). Total vitamin A is reported as the sum of retinol and beta-carotene (1/6 retinol equivalent).

### 2.4. Chemical Analysis: Feeds

Composited silage (3-week basis), concentrate (4-week basis) and grass samples (4-week basis) were analysed for general composition as previously described [6]. The fatty acid composition of feeds was determined using the procedure described by Sukhija and Palmquist [13], with the minor modification that toluene was used instead of benzene.

The concentrations of vitamin A and vitamin E in feeds were measured in a commercial laboratory (ALS Czech Republic, Na Harfe 336/9 Prague, Czech Republic). A liquid chromatography method with FLD detection was used (CZ_SOP_D06_04_206 (CSN EN 128 23-1, CSN EN 128 22)). Retinol and alpha tocopherol were determined by liquid chromatography, with a method uncertainty of 30%.

### 2.5. Calculations and Statistical Analysis

Daily grass DM intake was estimated based on the growth of the animals and their associated energy requirement [14] using the SAC Feedplan computer program (The Scottish Agricultural College, Aberdeen, UK). All statistical analyses were carried out using Genstat (21st edition, VSN International, Hemel Hempstead, England). For data pertaining to animal production and muscle chemical composition, group was the experimental unit, and production system was the source of variation. Identified individual fatty acids, except those associated with health-benefitting properties (less than or equal to 0.1% of the total fatty acid composition), were not considered for individual analysis. Selected nutritionally relevant fatty acid indices were calculated [15]. Muscle compositional data were initially analysed according to a split-plot design. The model had production system in the main plot and muscle and the muscle-by-production system interactions in the subplot. Because of a plethora of production system-by-muscle interactions, compositional data were re-analysed for individual muscles using the “production” model described above. Means were separated using a Fishers unprotected least significant difference test.

The possibility of classifying beef samples using the fatty acid profile (proportion of total fatty acids) according to the dietary background of the cattle or muscle type was examined via canonical discriminant analysis (CDA) in R [16]. For these analyses, individual animal data were used. A previous study [6] used a similar approach to discriminate between beef from different dietary treatments within an Irish controlled trial based on the fatty acid profile. Fatty acid data were first examined for non-detected values. If the proportion of non-detected FAMEs in a treatment or country group was <50%, non-detected values were replaced with the 0.5 limit of detection (LOD = 0.04 g/100 g of total fatty acids), and if the proportion of non-detected fatty acids was >50%, the fatty acid was regarded as non-detected for the full treatment group [17]. Statistical analysis was performed after correcting for non-detected values, and for analyses that required normally distributed data, only fatty acids with fewer than 15% non-detected values in each dietary treatment were included.

Two CDA models were developed. For Model A, the 4 production systems were considered and included all muscle types. For Model B, the 4 muscle types were considered and included all production systems. Prior to model building, data were randomly split (split ratio = 75:25) into training and validation datasets. The training set was used to develop the models, while the remainder was used for validation. The CDA generated a set of canonical discriminant functions (CDFs) that provide the best discrimination between dietary groups [18]. The relevance of each CDF was evaluated through Wilks’ lambda test. Models were assessed based on the classification results obtained from leave-one-out cross-validation (CV-LOO) and predictions resulting from applying the models to the test data sets. Splitting was carried out three times, and the results were displayed as the average of the three repeats.

## 3. Results

### 3.1. Feed Composition *(*Table 1*)*

The linseed oil-rich concentrate had a similar crude protein concentration but, as expected, a higher oil concentration and a higher proportion of C18:3 than the standard concentrate. The chemical compositions of the grass and grass silages used in the study were broadly similar but the DM digestibility and the proportion of C18:3 in the lipid of the grass tended to be higher than the grass silages. The forages had a higher proportion of C18:3 than the standard concentrate. The concentrations of vitamin E and beta-carotene tended to be highest in the finishing silage compared to the other feedstuffs.

### 3.2. Animal Production *(*Table 2*)*

Feed consumption data for the GRASS and CONC groups were previously reported (Reference [19] and are repeated here for comparison with feed consumption data for the other treatment groups. From January to April, mean daily silage DM consumption for the GRASS group was 5.3 kg animal^−1^. Mean daily silage DM consumption for the GSS and GSL groups was 3.5 and 3.7 kg animal^−1^, respectively. Concentrate DM intake for the latter two groups was 1.6 kg animal^−1^. From April to slaughter, mean daily grass DM consumption was estimated to be 9.3 kg animal^−1^ for the GRASS group. From April to housing, mean daily grass DM consumption was estimated to be 8.0 kg and 8.3 kg animal^−1^ for the GSS and GSL groups, respectively. From housing to slaughter, mean daily silage DM consumption for the GSS and GSL groups was 5.6 and 5.4 kg animal^−1^, respectively. The corresponding concentrate DM intake was 2.51 and 2.59 kg animal^−1^, respectively. For the CONC group, mean daily concentrate and silage DM consumption was 7.2 kg and 1.4 kg animal^−1^, respectively. The overall growth rate was lowest (*p* < 0.05) for the GRASS group, which was older (*p* < 0.05) at slaughter, and highest for the CONC group, which was younger at slaughter. The growth rate and age at slaughter of the GSS and GSL groups were intermediate, but the mean carcass weight, carcass fat classification and conformation score were similar for all groups.

### 3.3. General Chemical Composition *(*Table 3*)*

In a preliminary analysis of samples of cooked LM from the four treatment groups, the concentrations of vitamin A, retinol and beta-carotene were below the level of detection of the method (HPLC) used by the commercial laboratory. In cooked, pooled subcutaneous adipose tissue from animals in the four production systems, the concentration of vitamin A (sum of retinol and beta-carotene) was 67, 106, 74 and 64 (sed = 8.42) mg/kg for the CONC, GSS, GSL and GRASS groups, respectively.

With respect to aspects of the chemical composition, there were many interactions between production system and muscle type. In keeping with the objectives stated in the introduction, we focused first on the differences within the LM in animals finished on the high-concentrate diet and the three different modifications of this production system, then described the differences between those production systems for the other muscles examined. Stated differences are statistically significant at *p* < 0.05 or less. The moisture concentrations of LM, PM and ST muscles were similar for the four production systems. The moisture concentration of SUP was higher for the GSS group compared to the other groups, which did not differ. The protein concentrations of LM, PM and ST muscles were similar for the four production systems. The protein concentration of SUP was lower for the GSS group compared to the CONC and GRASS groups but similar to that of the GSL group.

The total lipid concentrations of LM, PM and ST were higher for the CONC group, compared to the other groups, which did not differ. The total lipid concentration of SUP was higher for GSS compared to GSL and GRASS, which did not differ, but similar to the CONC group. The energy concentration of muscle largely reflected the lipid concentration, as expected.

The cholesterol concentration of LM was higher for the CONC and GRASS groups, which did not differ compared to the GSS and GSL groups, which also did not differ. The cholesterol concentration of PM was lower for the CONC and GRASS groups (which did not differ) than the GSS group, which, in turn, was lower than GSL. The cholesterol concentration of ST was higher for CONC compared to the other groups, which did not differ, compared to the GSS and GSL groups, which also did not differ. The cholesterol concentration of SUP was lower for the CONC group compared to the GSS and GSL groups, which did not differ, but GSS was lower than GRASS, and GSL was similar to GRASS. The vitamin E concentrations of LM and SUP were lower for CONC compared to the other groups, which did not differ. The vitamin E concentrations of PM and ST were lower for the CONC group compared to the GSS and GSL groups, which did not differ, but the GRASS group was highest for PM but similar to GSS; and for ST but similar to GSL.

**Table 3:** Chemical compositions of muscles from heifers finished on concentrates ad libitum (CONC), grass silage and standard concentrate (GSS), grass silage and linseed concentrate (GSL) or grazed grass (Grass).

		Production System (PS)					
	Muscle ^1^	CONC	GSS	GSL	Grass	Sed ^2^	Significance
Moisture (g/kg)	SUP	638 ^a^	657 ^b^	644 ^a^	637 ^a^	6.0	**
	ST	619 ^a^	635 ^b^	634 ^b^	637 ^b^	7.2	0.06
	LM	631	628	623	614	8.8	NS
	PM	620	635	631	631	8.0	NS
Protein (g/kg)	SUP	296 ^b^	282 ^a^	294 ^ab^	302 ^b^	6.6	*
	ST	302	300	300	300	7.7	NS
	LM	290	298	303	291	8.9	NS
	PM	283	274	271	272	7.8	NS
Lipid (g/kg)	SUP	45 ^ab^	48 ^b^	38 ^a^	41 ^a^	3.3	*
	ST	54 ^b^	37 ^a^	40 ^a^	35 ^a^	5.4	**
	LM	61 ^b^	49 ^a^	43 ^a^	44 ^a^	4.8	**
	PM	85 ^b^	56 ^a^	57 ^a^	51 ^a^	5.8	***
Energy (KJ/100 g)	SUP	705	684	682	699	14.4	NS
	ST	757 ^b^	696 ^a^	703 ^a^	687 ^a^	19.3	**
	LM	750	732	727	745	18.7	NS
	PM	822 ^b^	734 ^a^	740 ^a^	730 ^a^	17.3	***
Cholesterol (mg/g)	SUP	0.29 ^a^	0.65 ^b^	0.73 ^bc^	0.78 ^c^	0.040	***
	ST	0.39 ^b^	0.31 ^a^	0.29 ^a^	0.30 ^a^	0.018	***
	LM	0.66 ^bc^	0.58 ^a^	0.61 ^ab^	0.69 ^c^	0.038	*
	PM	0.57 ^a^	0.66 ^b^	0.74 ^c^	0.57 ^a^	0.035	***
Vitamin E (mg/kg)	SUP	2.44 ^a^	6.14 ^b^	5.88 ^b^	6.47 ^b^	0.640	***
	ST	1.17 ^a^	2.71 ^b^	3.18 ^bc^	3.50 ^c^	0.337	***
	LM	1.35 ^a^	3.38 ^b^	3.25 ^b^	3.67 ^b^	0.301	***
	PM	1.99 ^a^	5.83 ^bc^	5.17 ^b^	7.02 ^c^	0.800	***

^1^ SUP = supraspinatus (chuck tender); ST = semitendinosus (eye of the round); LM = longissimus muscle (striploin); PM = psoas major (fillet). ^2^ sed = standard error of the difference. Means within a row with different letters differ significantly at *p* < 0.05. NS = not significant; * = *p* < 0.05; ** = *p* < 0.01; *** = *p* < 0.001.

### 3.4. Categories of Fatty Acids and Selected Nutritional Indices *(*Table 4*)*

The total saturated fatty acid concentration in LM was similar for CONC and GSS but higher than GSL and GRASS. The total saturated fatty acid concentration in PM was higher for the CONC group compared to the other groups, which did not differ. The total saturated fatty acid concentration in ST was similar for CONC, GSS and GSL but higher than GRASS. The total saturated fatty acid concentration in SUP was similar for CONC and GSS but higher than GSL, while GSL and GRASS were similar.

The total monounsaturated fatty acid concentrations in LM, PM and ST were higher for the CONC group compared to the other groups, which did not differ. The total monounsaturated fatty acid concentration in SUP was similar for CONC and GSS but higher than GSL, while GRASS was similar to CONC and GSS.

The total polyunsaturated fatty acid concentrations in LM, PM and ST were higher for the CONC group compared to the other groups, which did not differ. The total polyunsaturated fatty acid concentration in SUP was lower for the CONC and GSL groups, which did not differ, compared to the GSS and GRASS groups, which also did not differ. The concentration of n-3 polyunsaturated fatty acids in LM and ST was higher for the GRASS group compared to the other groups, which did not differ.

The total n-3 polyunsaturated fatty acid concentration in LM was similar in CONC and GSL; both were higher than GSS and lower than GRASS. The total n-3 polyunsaturated fatty acid concentration in PM was lower for the CONC group compared to the other groups, which did not differ. The total n-3 polyunsaturated fatty acid concentration in ST was similar for CONC and GSS; both were lower than GSL, which, in turn, was lower than GRASS. The total n-3 polyunsaturated fatty acid concentration in SUP was lower in CONC compared to GSS and GSL, which did not differ and were, in turn, lower than GRASS.

The total n-6 polyunsaturated fatty acid concentrations in LM, PM and ST were higher for the CONC group compared to the GSS and GSL groups, which did not differ and were, in turn, higher than GRASS. The total n-6 polyunsaturated fatty acid concentration in SUP was higher for the CONC group compared to the other groups, which did not differ.

The total *trans* fatty acid concentrations in LM, PM and ST were similar for all groups. The total *trans* fatty acid concentration in SUP was lower in the CONC group compared to the GSS and GSL groups, which did not differ, while GSL was lower than GRASS.

The polyunsaturated-to-saturated fatty acid ratio in LM was similar for CONC, GSL and GRASS but higher than GSS. The polyunsaturated-to-saturated fatty acid ratio in PM, was lower for the CONC group compared to the GSS and GSL groups, which did not differ and were, in turn, lower than GRASS. The polyunsaturated-to-saturated fatty acid ratio in ST was similar for CONC, GSL and GSS, but GRASS was higher than GSS and GSL. The polyunsaturated-to-saturated fatty acid ratio in SUP was similar for the CONC and GSS groups which did not differ, but both were lower than the GSL and GRASS groups, which also did not differ.

The n-6-to-n-3 polyunsaturated fatty acid ratio in LM was similar for the CONC and GSS groups; both were higher than the GSL group, which, in turn, was higher than GRASS. The n-6-to-n-3 polyunsaturated fatty acid ratio in PM was higher for the CONC group compared to the other groups, which did not differ. The n-6-to-n-3 polyunsaturated fatty acid ratio in ST was higher for CONC than GSS, which was higher than GSL, which, in turn, was higher than GRASS. The n-6-to-n-3 polyunsaturated fatty acid ratio in SUP was higher for the CONC group compared to the other groups, which did not differ.

The atherogenic indices for LM, PM and SUP were higher for GSS than CONC, which was higher than GSL and GRASS, which did not differ. The atherogenic index for ST was higher for GSS than GSL, which was higher than CONC and GRASS, which did not differ.

The thrombogenic index for LM was higher for the GSS group compared to the CONC group, which was higher than the GSL group, which, in turn, was higher than the GRASS group. The thrombogenic index for PM was higher for CONC than GSS, which was higher than GSL, which, in turn, was higher than GRASS. The thrombogenic index for ST was similar for GSS and GSL; both were higher than CONC, which, in turn, was higher than GRASS. The thrombogenic index for SUP was similar for CONC and GSS; both were higher than GSL, which was higher than GRASS.

The hypocholesterolaemic–hypercholesterolaemic indices for LM, PM and SUP were lower for the GSS group than the CONC group, which, in turn, was lower than the GSL and GRASS groups, which did not differ. The hypocholesterolaemic–hypercholesterolaemic index for ST was lower for GSS than GSL, which was lower than CONC and GRASS, which did not differ.

**Table 4:** Categories of fatty acids ^1^ (mg/100 g cooked muscle) and nutritional indices ^2^ of muscles from heifers finished on concentrates ad libitum (CONC), grass silage and standard concentrate (GSS), grass silage and linseed concentrate (GSL) or grazed grass (Grass).

		Treatment					
	Muscle ^3^	CONC	GSS	GSL	Grass	Sed ^4^	Significance
SFA	SUP	1848 ^bc^	2092 ^c^	1483 ^a^	1583 ^ab^	151.2	***
	ST	2157 ^b^	1697 ^ab^	1769 ^ab^	1505 ^a^	243.0	0.07
	LM	2495 ^b^	2194 ^ab^	1808 ^a^	1823 ^a^	209.8	**
	PM	3731 ^b^	2612 ^a^	2515 ^a^	2269 ^a^	272.6	***
MUFA	SUP	2218 ^b^	2149 ^b^	1773 ^a^	1912 ^ab^	166.2	*
	ST	2858 ^b^	1759 ^a^	1901 ^a^	1695 ^a^	276.6	***
	LM	2990 ^b^	2376 ^a^	2202 ^a^	2245 ^a^	244.4	**
	PM	4064 ^b^	2464 ^a^	2653 ^a^	2308 ^a^	274.2	***
PUFA	SUP	284 ^a^	321 ^b^	282 ^a^	322 ^b^	17.1	*
	ST	310 ^b^	214 ^a^	235 ^a^	240 ^a^	20.0	***
	LM	327 ^b^	228 ^a^	217 ^a^	259 ^a^	21.5	***
	PM	399 ^b^	324 ^a^	325 ^a^	330 ^a^	24.1	**
n-3 PUFA	SUP	21 ^a^	88 ^b^	90 ^b^	117 ^c^	6.5	***
	ST	47 ^a^	52 ^a^	70 ^b^	112 ^c^	7.0	***
	LM	67 ^b^	45 ^a^	75 ^b^	107 ^c^	7.6	***
	PM	60 ^a^	115 ^b^	106 ^b^	127 ^b^	11.0	***
n-6 PUFA	SUP	209 ^b^	151 ^a^	157 ^a^	152 ^a^	7.7	***
	ST	176 ^c^	123 ^b^	112 ^b^	97 ^a^	5.6	***
	LM	176 ^c^	116 ^b^	107 ^ab^	100 ^a^	7.3	***
	PM	287 ^c^	174 ^b^	178 ^b^	154 ^a^	9.7	***
Trans FA	SUP	70 ^a^	200 ^bc^	156 ^b^	235 ^c^	25.3	***
	ST	103	84	130	159	32.9	NS
	LM	152	123	113	168	24.6	NS
	PM	266 ^ab^	193 ^a^	203 ^ab^	272 ^b^	37.2	0.08
PUFA:SFA	SUP	0.16 ^a^	0.16 ^a^	0.20 ^b^	0.21 ^b^	0.009	***
	ST	0.15 ^ab^	0.13 ^a^	0.14 ^a^	0.17 ^b^	0.012	*
	LM	0.14 ^b^	0.10 ^a^	0.13 ^b^	0.14 ^b^	0.009	***
	PM	0.11 ^a^	0.13 ^b^	0.13 ^b^	0.15 ^c^	0.008	***
n-6:n-3 PUFA	SUP	11.5 ^a^	1.70 ^ab^	1.71 ^a^	1.33 ^a^	1.048	***
	ST	4.14 ^d^	2.45 ^c^	1.67 ^b^	0.89 ^a^	0.304	***
	LM	2.71 ^c^	2.83 ^c^	1.47 ^b^	1.00 ^a^	0.187	***
	PM	5.81 ^b^	1.53 ^a^	1.88 ^a^	1.26 ^a^	0.449	***
AI	SUP	0.62 ^b^	0.68 ^c^	0.52 ^a^	0.51 ^a^	0.020	***
	ST	0.65 ^a^	0.76 ^c^	0.71 ^b^	0.61 ^a^	0.024	***
	LM	0.66 ^b^	0.75 ^c^	0.62 ^a^	0.60 ^a^	0.019	***
	PM	0.72 ^b^	0.76 ^c^	0.65 ^a^	0.64 ^a^	0.019	***
TI	SUP	1.39 ^c^	1.33 ^c^	1.09 ^b^	1.03 ^a^	0.032	***
	ST	1.23 ^b^	1.41 ^c^	1.33 ^c^	1.06 ^a^	0.048	***
	LM	1.30 ^c^	1.51 ^d^	1.20 ^b^	1.11 ^a^	0.030	***
	PM	1.51 ^d^	1.43 ^c^	1.35 ^b^	1.27 ^a^	0.034	***
HH	SUP	1.83 ^b^	1.61 ^a^	2.02 ^c^	2.05 ^c^	0.057	***
	ST	1.68 ^c^	1.45 ^a^	1.56 ^b^	1.75 ^c^	0.049	***
	LM	1.65 ^b^	1.45 ^a^	1.70 ^bc^	1.76 ^c^	0.044	***
	PM	1.55 ^b^	1.46 ^a^	1.67 ^c^	1.70 ^c^	0.040	***

^1^ SFA = total saturated fatty acids; MUFA = total monounsaturated fatty acids; PUFA = total polyunsaturated fatty acids; Trans = total trans fatty acids. ^2^ AI = atherogenic index; TI = thrombogenic index; HH = hypo:hypercholesterolaemic index (C18:1 + PUFA) ÷ (C12:0 + C14:0 + C16:0). ^3^ SUP = supraspinatus (chuck tender); ST = semitendinosus (eye of the round); LM = longissimus muscle (striploin); PM = psoas major (fillet). ^4^ sed = standard error of the difference. Means within a row with different letters differ significantly at *p* < 0.05. NS = not significant; * = *p* < 0.05; ** = *p* < 0.01; *** = *p* < 0.001.

### 3.5. Individual Saturated Fatty Acids *(*Table 5*)*

Of the important fatty acids, the C14:0 concentration in LM and SUP was similar for CONC and GSS but higher than GSL and GRASS, which did not differ. The C14:0 concentration in PM was higher for CONC than GSS and GSL, which did not differ, and GSS was higher than GRASS, but GSL and GRASS did not differ. The C14:0 concentration in ST was higher for CONC compared to the other groups, which did not differ.

The C16:0 concentration in LM was similar for CONC and GSS; both were higher than GRASS, while GSS was similar to GSL, and GSL was similar to GRASS. The C16:0 concentrations in PM and ST were higher for CONC compared to the other groups, which did not differ. The C16:0 concentration in SUP was similar for CONC and GSS, but both were higher than GSL and GRASS, which did not differ.

The C18:0 concentration in LM and ST was similar for all groups. The C18:0 concentration in PM was higher for CONC compared to the other groups, which did not differ. The C18:0 concentration in SUP was similar for CONC, GSL and GRASS, and GSS was higher than CONC and GSL but similar to GRASS.

**Table 5:** Concentrations of individual saturated fatty acids (mg/100 g cooked muscle) of muscles from heifers finished on concentrates ad libitum (CONC), grass silage and standard concentrate (GSS), grass silage and linseed concentrate (GSL) or grazed grass (Grass).

		Treatment					
	Muscle ^1^	CONC	GSS	GSL	Grass	Sed ^2^	Significance
C14:0	SUP	113 ^b^	131 ^b^	68 ^a^	77 ^a^	10.6	**
	ST	159 ^b^	121 ^a^	111 ^a^	86 ^a^	18.5	**
	LM	164 ^b^	143 ^b^	100 ^a^	103 ^a^	13.9	***
	PM	262 ^c^	176 ^b^	141 ^ab^	129 ^a^	19.6	***
C15:0	SUP	11 ^a^	22 ^b^	13 ^a^	17 ^a^	3.2	**
	ST	22 ^b^	7 ^a^	16 ^ab^	8 ^a^	4.9	*
	LM	21 ^b^	18 ^b^	5 ^a^	9 ^a^	3.7	***
	PM	40 ^b^	32 ^ab^	26 ^a^	27 ^a^	4.9	*
C16:0	SUP	1058 ^b^	1115 ^b^	773 ^a^	804 ^a^	77.5	***
	ST	1378 ^b^	1004 ^a^	1045 ^a^	828 ^a^	140.5	**
	LM	1497 ^c^	1328 ^bc^	1088 ^ab^	1063 ^a^	127.6	**
	PM	2115 ^b^	1413 ^a^	1338 ^a^	1143 ^a^	150.4	***
C17:0	SUP	50	49	37	43	5.5	NS
	ST	56 ^b^	46 ^b^	31 ^a^	39 ^a^	7.3	*
	LM	60 ^b^	49 ^ab^	40 ^a^	43 ^a^	5.8	**
	PM	101 ^b^	69 ^a^	69 ^a^	67 ^a^	7.7	***
C17:0 i+ C16:1 t9	SUP	9 ^b^	21 ^c^	3 ^a^	4 ^a^	2.3	***
	ST	16 ^b^	8 ^ab^	6 ^a^	16 ^b^	4.5	*
	LM	17 ^b^	18 ^b^	6 ^a^	13 ^ab^	3.5	**
	PM	34 ^b^	35 ^b^	25 ^a^	28 ^ab^	4.4	*
C18:0	SUP	616 ^a^	736 ^b^	573 ^a^	626 ^ab^	57.7	*
	ST	542	502	563	543	79.2	NS
	LM	726	654	575	604	64.9	NS
	PM	1197 ^b^	895 ^a^	940 ^a^	900 ^a^	94.9	**

^1^ SUP = supraspinatus (chuck tender); ST = semitendinosus (eye of the round); LM = longissimus muscle (striploin); PM = psoas major (fillet). ^2^ sed = standard error of the difference. Means within a row with different letters differ significantly at *p* < 0.05. NS = not significant; * = *p* < 0.05; ** = *p* < 0.01; *** = *p* < 0.001.

### 3.6. Individual Monounnsaturated Fatty Acids *(*Table 6*)*

Of the important fatty acids, the C16:1c9 concentration in LM was similar for CONC and GSS, and CONC was higher than GSL and GRASS, which did not differ, while GSS, GSL and GRASS were similar. The C16:1c9 concentrations in PM and ST were higher for CONC compared to the other groups, which did not differ. The C16:1c9 concentration in SUP was similar for CONC and GSS and higher than GSL and GRASS, which did not differ.

The C18:1c9 concentrations in LM, PM and ST were higher for CONC compared to the other groups, which did not differ. The C18:1c9 concentration in SUP was similar for CONC and GSS, and CONC was higher than GSL and GRASS, which did not differ.

The C18:1c11 concentrations in LM, PM, ST and SUP were higher for CONC compared to the other groups, which did not differ. The C18:1t11 concentration in LM was lower for CONC than GSS and GSL, which did not differ and which, in turn, were lower than GRASS. The C18:1t11 concentration in PM was similar for CONC, GSL and GSS, which were lower than GRASS. The C18:1t11 concentration in ST was similar for CONC and GSS, and CONC was lower than GSL and GRASS, which did not differ, while GSS was similar to GSL. The C18:1t10concentrations in LM, PM and ST were higher for CONC compared to the other groups, which did not differ, while the C18:1t10 concentration in SUP did not differ between groups.

**Table 6:** Concentration of individual monounsaturated fatty acids (mg/100 g cooked muscle) of muscles from heifers finished on concentrates ad libitum (CONC), grass silage and standard concentrate (GSS), grass silage and linseed concentrate (GSL) or grazed grass (Grass).

		Treatment					
	Muscle ^1^	CONC	GSS	GSL	Grass	Sed ^2^	Significance
C14:1	SUP	25 ^b^	27 ^b^	17 ^a^	17 ^a^	2.8	***
	ST	63 ^c^	39 ^b^	29 ^b^	9 ^a^	7.6	***
	LM	46 ^c^	40 ^bc^	28 ^a^	29 ^ab^	5.8	**
	PM	57 ^c^	34 ^b^	29 ^ab^	20 ^a^	5.7	***
C16:1 t10 to 12	SUP	3 ^a^	13 ^b^	16 ^b^	21 ^c^	1.9	***
	ST	9 ^b^	1 ^a^	3 ^ab^	7 ^b^	3.4	*
	LM	12 ^b^	3 ^a^	1 ^a^	2 ^ab^	1.7	***
	PM	24 ^b^	18 ^b^	1 ^a^	8 ^a^	3.7	***
C16:1 c9+ C17:0 ai	SUP	166 ^b^	160 ^b^	112 ^a^	121 ^a^	12.3	***
	ST	306 ^b^	169 ^a^	153 ^a^	126 ^a^	27.0	***
	LM	250 ^b^	227 ^ab^	185 ^a^	192 ^a^	21.8	*
	PM	316 ^b^	199 ^a^	191 ^a^	158 ^a^	23.1	***
C17:1 c9	SUP	31 ^b^	35 ^b^	13 ^a^	13 ^a^	2.0	***
	ST	25 ^b^	18 ^b^	11 ^a^	32 ^c^	3.5	***
	LM	28 ^a^	27 ^a^	32 ^b^	34 ^b^	1.4	***
	PM	32 ^a^	28 ^a^	44 ^b^	46 ^b^	2.8	***
C18:1 t9	SUP	2 ^a^	10 ^c^	8 ^b^	9 ^bc^	1.1	***
	ST	5	3	6	1	2.5	NS
	LM	12 ^b^	1 ^a^	1 ^a^	1 ^a^	1.2	***
	PM	19 ^c^	4 ^b^	1 ^a^	1 ^a^	1.8	***
C18:1 t10	SUP	9	6	5	6	2.4	NS
	ST	14 ^b^	1 ^a^	1 ^a^	1 ^a^	2.5	***
	LM	18 ^c^	7 ^b^	1 ^a^	1 ^a^	2.3	***
	PM	30 ^b^	3 ^a^	1 ^a^	1 ^a^	4.0	***
C18:1 t11	SUP	39 ^a^	86 ^bc^	62 ^ab^	110 ^c^	12.8	***
	ST	41 ^a^	67 ^ab^	85 ^bc^	112 ^c^	16.6	**
	LM	39 ^a^	78 ^b^	76 ^b^	126 ^c^	14.4	***
	PM	102 ^a^	114 ^a^	128 ^a^	182 ^b^	19.5	**
C18:1 c9	SUP	1837 ^b^	1670 ^ab^	1408 ^a^	1464 ^a^	131.0	**
	ST	2221 ^b^	1398 ^a^	1545 ^a^	1337 ^a^	218.4	***
	LM	2387 ^b^	1905 ^a^	1774 ^a^	1779 ^a^	198.7	*
	PM	3263 ^b^	1959 ^a^	2145 ^a^	1805 ^a^	217.9	***
C18:1 c11	SUP	84 ^b^	63 ^a^	52 ^a^	59 ^a^	5.3	***
	ST	113 ^b^	54 ^a^	53 ^a^	51 ^a^	7.5	***
	LM	98 ^b^	68 ^a^	61 ^a^	66 ^a^	6.7	***
	PM	128 ^b^	66 ^a^	69 ^a^	64 ^a^	6.7	***
C18:1 c13	SUP	19 ^b^	16 ^ab^	14 ^a^	14 ^a^	2.0	*
	ST	42 ^c^	6 ^ab^	10 ^b^	2 ^a^	4.0	***
	LM	30 ^c^	17 ^b^	15 ^b^	1 ^a^	3.5	***
	PM	35 ^c^	14 ^b^	12 ^ab^	5 ^a^	3.8	***
C18:1 t16	SUP	1 ^a^	12 ^b^	11 ^b^	11 ^b^	1.2	***
	ST	1	1	3	2	2.1	NS
	LM	8 ^b^	1 ^a^	1 ^a^	1 ^a^	6.8	***
	PM	12	9	12	8	3.6	NS

^1^ SUP = supraspinatus (chuck tender); ST = semitendinosus (eye of the round); LM = longissimus muscle (striploin); PM = psoas major (fillet). ^2^ sed = standard error of the difference. Means within a row with different letters differ significantly at *p* < 0.05. NS = not significant; * = *p* < 0.05; ** = *p* < 0.01; *** = *p* < 0.001.

### 3.7. Individual Polyunsaturated Fatty Acids *(*Table 7*)*

Of the important fatty acids, the C18:2c9,c12 concentration in LM was higher for CONC than GSS and GSL, which did not differ, and GSS was higher than GRASS, while GSL and GRASS were similar. The C18:2c9,c12 concentrations in PM and ST were higher for CONC than GSS and GSL, which did not differ, which, in turn, were higher than GRASS. The C18:2c9,c12 concentration in SUP was higher for CONC compared to the other groups, which did not differ.

The C18:3c9,c12,c15 concentrations in LM and SUP were lower in CONC than in GSS and GSL, which did not differ and were, in turn, lower than GRASS. The C18:3c9,c12,c15 concentrations in PM and ST were lower for CONC than GSS, which, in turn, was lower than GSL and GRASS, which did not differ.

The C18:2c9,t11 concentration in LM was lower in CONC than GSS and GSL, which did not differ, and GSL was higher than GRASS but similar to GSS. The C18:2c9,t11 concentration in PM was similar in CONC, GSL and GRASS, and all were higher than GSS. The C18:2c9,t11 concentration in ST did not differ between groups, while the C18:2c9,t11 concentration in SUP was lower in CONC than GSS and GSL, which did not differ and were, in turn, lower than GRASS.

The C20:4 concentration in LM did not differ between groups. The C20:4 concentration in PM was higher in CONC compared to the other groups, and GSS was similar to GSL and GRASS, while GSL was higher than GRASS. The C20:4 concentration in ST was higher in CONC than GSS, which, in turn, was higher than GSL and GRASS, which did not differ. The C20:4 concentration in SUP was higher in CONC than the other groups, and GSS was lower than GSL and GRASS, which did not differ.

The C20:5 concentration in LM was higher in CONC than GSS but similar to GSL and GRASS, and GSS was similar to GSL, both of which were lower than GRASS. The C20:5 concentrations in PM and SUP were lower in CONC than GSS, which was lower than GSL, which, in turn, was lower than GRASS. The C20:5 concentrations in ST were similar for CONC, GSS and GSL, and all were lower than GRASS.

The C22:5 concentration was generally below the level of detection for CONC, GSS and GSL for all muscles. For GRASS, the C22:5 concentration averaged 31, 35, 37 and 3 mg/100 g for LM, PM, ST and SUP, respectively. The C22:6 concentration was below the level of detection in most analysed samples.

**Table 7:** Concentrations of individual polyunsaturated fatty acids (mg/100 g cooked muscle) of muscles from heifers finished on concentrates ad libitum (CONC), grass silage and standard concentrate (GSS), grass silage and linseed concentrate (GSL) or grazed grass (Grass).

		Treatment					
	Muscle ^1^	CONC	GSS	GSL	Grass	Sed ^2^	Significance
C16:2 c9,c12	SUP	40 ^c^	28 ^b^	1 ^a^	1 ^a^	2.6	***
	ST	60 ^b^	1 ^a^	1 ^a^	1 ^a^	4.0	***
	LM	46 ^c^	37 ^c^	10 ^a^	23 ^b^	6.1	***
	PM	10 ^ab^	4 ^a^	20 ^b^	11 ^ab^	5.8	0.08
C18:2 c9,12	SUP	147 ^b^	109 ^a^	105 ^a^	102 ^a^	5.9	***
	ST	128 ^c^	89 ^b^	82 ^b^	68 ^a^	5.1	***
	LM	133 ^c^	88 ^b^	80 ^ab^	72 ^a^	5.9	***
	PM	219 ^c^	135 ^b^	136 ^b^	118 ^a^	8.2	***
C18:3 c9,12,15	SUP	19 ^a^	41 ^b^	46 ^b^	60 ^c^	3.3	***
	ST	23 ^a^	34 ^b^	46 ^c^	43 ^c^	2.9	***
	LM	22 ^a^	37 ^b^	40 ^b^	48 ^c^	2.6	***
	PM	41 ^a^	54 ^b^	67 ^c^	74 ^c^	4.1	***
C18:2 c9,t11 (CLA)	SUP	13 ^a^	26 ^b^	22 ^b^	36 ^c^	4.2	**
	ST	21	15	23	30	6.1	NS
	LM	16 ^a^	28 ^bc^	34 ^c^	23 ^ab^	5.2	*
	PM	38 ^b^	27 ^a^	41 ^b^	49 ^b^	5.7	**
C20:3 c8,c11, c14	SUP	16 ^b^	10 ^a^	11 ^a^	11 ^a^	1.4	***
	ST	10 ^b^	1 ^a^	1 ^a^	1 ^a^	1.3	***
	LM	14 ^b^	1 ^a^	1 ^a^	1 ^a^	0.4	***
	PM	19 ^b^	1 ^a^	1 ^a^	1 ^a^	0.9	***
C20:4 c5,8,11, 14	SUP	47 ^c^	31 ^a^	40 ^b^	37 ^b^	1.7	***
	ST	37 ^c^	34 ^b^	30 ^a^	29 ^a^	1.4	***
	LM	28	28	27	28	2.6	NS
	PM	49 ^c^	40 ^ab^	41 ^b^	36 ^a^	2.0	***
C20:5 c5,8,11, 14,17	SUP	2 ^a^	15 ^b^	22 ^c^	27 ^d^	1.2	***
	ST	14 ^a^	17 ^a^	19 ^a^	25 ^b^	2.8	**
	LM	11 ^bc^	3 ^a^	5 ^ab^	16 ^c^	3.1	***
	PM	2 ^a^	13 ^b^	21 ^c^	29 ^d^	3.1	***

^1^ SUP = supraspinatus (chuck tender); ST = semitendinosus (eye of the round); LM = longissimus muscle (striploin); PM = psoas major (fillet). ^2^ sed = standard error of the difference. Means within a row with different letters differ significantly at *p* < 0.05. NS = not significant; * = *p* < 0.05; ** = *p* < 0.01; *** = *p* < 0.001.

### 3.8. Discrimination According to Dietary Treatment

**Figure 1:**
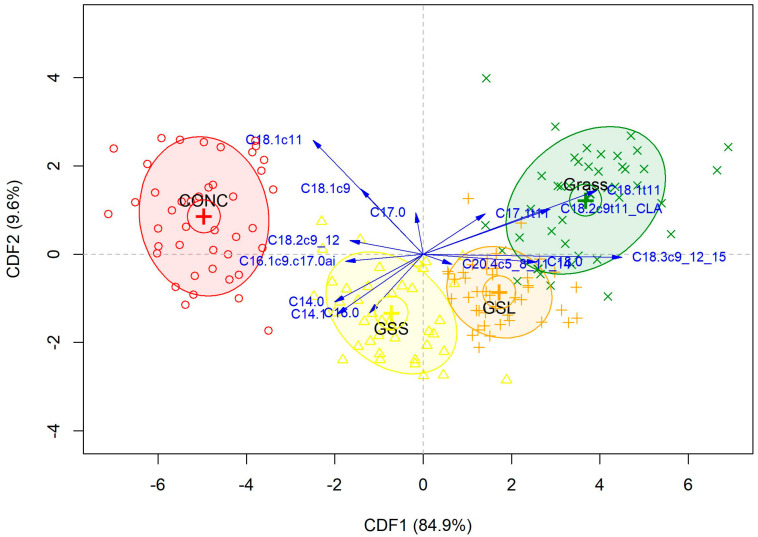
Canonical score and structure coefficient plot for the 1st and 2nd canonical discriminant functions (CDF1 and CDF2) of model A (first repeat) with beef from heifers finished on concentrates ad libitum (CONC), grass silage and standard concentrate (GSS), grass silage and linseed concentrate (GSL) or grazed grass (Grass).

Model A was developed to discriminate between the dietary treatments without considering the different beef muscles. For this model, three CDFs were necessary for discrimination. On average, the first CDF explained 87.08% of the variance, the second CDF explained 8.67% and the third CDF explained 4.24%. The score plot for CDF1 vs. CDF2 obtained for the first repeat is shown in Figure 1.

The average classification results from the three repeats of CV-LOO are displayed in Table 8. The CDA model distinguished between the four dietary treatments with an overall accuracy (the ratio between the number of correctly classified samples and the total number of samples) of 91.6%. The highest sensitivity was achieved for classification of CONC and GSL samples. The model correctly classified 42.0 out of 42 CONC samples (sensitivity = 100.0%) 38.0 out of 42 GSL samples (sensitivity = 90.5%). The numbers of correctly classified GSS and GRASS samples were lower (36.3 out of 41 with sensitivity = 88.6% and 39.3 out of 45 with sensitivity = 87.4%, respectively). Validation with the test set of samples clarified the ability of the model to discriminate between the dietary treatments, with overall accuracy = 87.1%. On average, the model correctly classified 14.0, 11.7, 12.0 and 12.0 out of 14, 14, 14 and 15 samples from CONC, GSS, GSL and GRASS, respectively.

### 3.9. Discrimination According to Muscle Type

**Figure 2:**
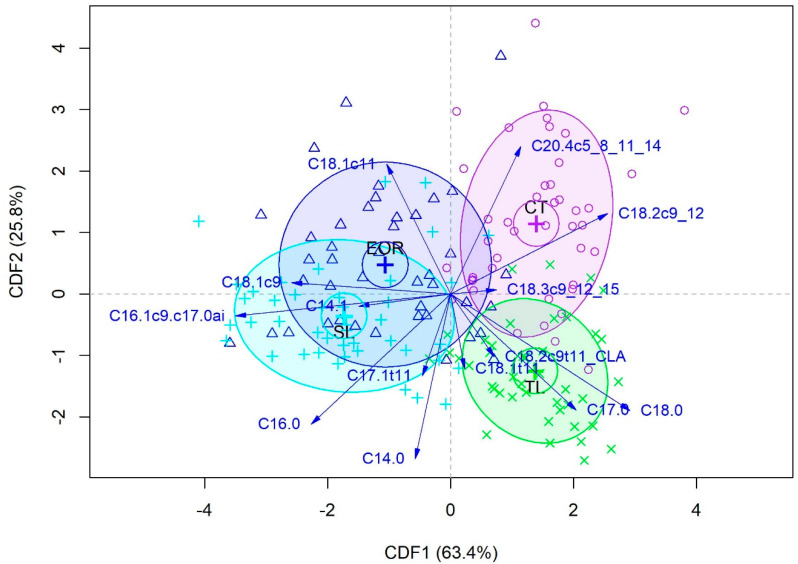
Canonical score and structure coefficient plot for the 1st and 2nd canonical discriminant functions (CDF1 and CDF2) of Model B (first repeat) with chuck tender (CT), eye of round (EOR), striploin (SL) and tenderloin (TL) muscles.

Model B was developed to discriminate between the different beef muscles without considering dietary treatment. For this model, three CDF were necessary for the discrimination. On average, the first CDF explained 58.23% of the variance, the second CDF explained 29.56% and the third CDF explained 12.19%. The score plot for CDF1 vs. CDF2 obtained for the first repeat is shown in Figure 2.

The average classification results from the three repeats of CV-LOO are displayed in Table 8. The CDA model distinguished between the four muscles with an overall accuracy of 75.4%. The highest sensitivity was achieved for classification of CT and TL samples. The model correctly classified 34.0 out of 43 CT samples (sensitivity = 79.1%) and 37.3 out of 43 TL samples (sensitivity = 86.8%). The numbers of correctly classified EOR and SL samples were lower (26.7 out of 42 with sensitivity = 63.5% and 31.07 out of 43 with sensitivity = 72.1%, respectively). Validation with the test set of muscles clarified the ability of the model to discriminate between them, with overall accuracy = 77.4%. On average, the model correctly classified 11.0, 9.0, 11.7 and 11.7 out of 14 samples each from CT, EOR, SL and TL, respectively.

## 4. Discussion

### 4.1. Context

An early-maturing breed heifer production system was chosen, since Spring-born heifers can reach a commercially relevant carcass weight (260 kg) at less than 20 months of age when grazing pasture in a temperate environment [20]. They are therefore particularly suited to the production of beef from a grazed grass and ensiled grass dietary regimen without concentrate supplementation, and the target carcass weight was achieved from pasture. The animals were allowed to express their growth potential on both the concentrate and grass diets. The concentrate-based production system also represented the extreme comparison with a lifetime grass-based production system. Heifers are also often housed towards the end of the grazing season due to prevailing weather conditions and offered a finishing diet based on grass silage ad libitum and sufficient supplementary concentrates to reach the target slaughter weight [20]. This production system was represented by GSS and has been marketed as ”grass-based”. In recognition of the expected loss of omega-3 PUFA and conjugated linoleic acid (CLA), when cattle were moved from pasture to the indoor ration [21], and to determine if the diet of non-grazing cattle could be manipulated such that muscle from “indoor” cattle could have the same fatty acid profile as their grazing counterparts, the concentrate was fortified with linseed in GSL. The amount of ruminally-protected linseed meal (approximately 80% protection of the linseed oil according to the supplier) was chosen based on an assumed supply of linolenic acid to the small intestine of GRASS animals. Linseed oil was also supplemented to match the assumed level of consumption of GRASS animals to allow for ruminal production of bioactive hydrogenation intermediates such as conjugated linoleic acid (below). All animals were slaughtered when the target carcass weight was expected to be achieved. This resulted in different ages at slaughter, but all groups had a similar mean carcass weight, as planned. The growth rates in the different periods of the experiment were as expected based on the imposed management strategies, and the carcass conformation and average fatness scores were largely similar.

Our study expanded the scope of our previous study on the enhancement of the nutritional value of beef [22] by considering, in addition to the LM, which is usually chosen as an “indicator” muscle, a high-value muscle (PM, the commercial fillet) and a lower value muscle (ST, commercial “eye of the round”). These muscles also represent a range in IMF concentrations. The SUP muscle (commercial “chuck tender”) was chosen as a muscle from the forequarter of the carcass, as such muscles are generally minced and therefore have a lower commercial value. We chose to carry out our experiment on cooked muscle because consumers generally do not eat uncooked muscle. The sous vide method of cooking was chosen because it is growing in popularity due to its convenience but also avoids potential damage to the fatty acid profile in the outer edges of the muscle that can occur during grilling or frying. We acknowledge that the data that we generated may also be influenced by the particular method of cooking chosen. Our preliminary experiment (Supplementary Table S1) demonstrated that the sous vide method of cooking is largely benign with respect to the fatty acid profile and that it results in a concentration of the beneficial fatty acids primarily due to the loss of moisture due to cooking. A similar effect has been reported for sheep meat [23].

Within the EU, a nutrition claim made with respect to a food must comply with Nutrition and health claims Regulation 1924/2006 [24,25]. According to Regulation 1924/2006, red meats can be described as a ‘source of’ or ‘high in’ a nutrient. Regulation 1924/2006 also states that “a claim that a food is a ‘source of’ may only be made where the product contains at least a significant amount, i.e., 15% of the recommended dietary allowance (RDA)” and that “a claim that a food is ‘high in’ may only be made where the product contains at least twice the value of ‘source of’”. It also states that “a claim that a food ‘contains’ a nutrient or another substance may only be made when specific conditions are not laid down in the Regulation”. The muscle composition data were interpreting in terms of the possibility of making a nutritional claim for combinations of beef production system and muscle type.

### 4.2. General Chemical Composition

There is very limited information in the literature on the influence of the diet of cattle on the vitamin A and/or β-carotene concentrations in beef. Uncooked muscle from steers finished and/or raised on forage diets or forage-based production systems contained more β-carotene than beef from steers finished and/or raised on concentrate diets or concentrate-based production systems [26]. Our study indicates that at the observed IMF concentrations and after cooking, the inclusion of grass in the ration of beef cattle would have little effect on the vitamin A content in beef.

There appears to be no RDA for cholesterol, and generally, there is little significant difference in the content of cholesterol between beef from grass-based and concentrate-based production systems [26,27]. Doyle et al. [27] reported a lower cholesterol concentration in uncooked LM from cattle finished on a ration similar to GSS compared to a ration similar to CONC and concluded that the absolute difference was unlikely to affect human plasma cholesterol level. The data in the present study are largely consistent with the above reports, i.e., minor effects, with the exception of SUP, where GRASS resulted in a 2.7-fold increase when compared to CONC. However, the implications of consuming SUP from grass-fed cattle for human heath are unknown. There are few reports on the cholesterol concentrations in different beef muscles from animals on the same dietary regimen. In this study, the concentration of cholesterol was consistently lower in the ST muscle compared to LM, which is consistent with a study by Alfaia et al. [28]. These authors suggested that this might reflect differences in fibre type composition and/or the higher muscle lipid concentration in LM compared to the ST muscle, as was seen in the present study.

In general, grass feeding and/or inclusion of grass in the ration of beef cattle increases the vitamin E concentration in beef [2]. This was also the case in the present study, where the trend was for higher vitamin E concentrations in muscle from cattle in the three grass-based production systems, with little difference due to diet in the final finishing phase when cattle were pre-grazed. It is noteworthy that the biggest increase (3.5-fold) due to GRASS was for PM. This value (0.7 mg/100 g) would still be below the level (2.25 mg/100 g, 15% of RDA) required for PM from GRASS to be labelled a ‘source of’ vitamin E [25].

The generally higher IMF concentrations in all muscles from the CONC group despite the similar carcass fatness score may reflect the different substrate requirements and supplies (acetate vs. propionate from grass and concentrate diets) for lipid synthesis in subcutaneous and intramuscular adipocytes. The mean difference between muscles in IMF concentration was as expected, i.e., highest for PM and lowest for ST (and SUP) with LM as the intermediate [4]. Based on the EU definition of a “low-fat” food, i.e., total fatty acids <3 g/100 g solid [25], none of the muscles produced in this study could be labelled as low-fat.

Presentation of fatty acid data expressed as a proportion of total fatty acids can allow for a more complete comparison with the literature. However, from a product labelling and marketing perspective, concentration data are more relevant for some variables, while for others, proportional data are more relevant. Accordingly, relevant fatty acid data are also presented in proportion form in Supplementary Table S2. The concentrations of total saturated fatty acids, total monounsaturated fatty acids and total polyunsaturated fatty acids generally reflect the IMF concentration, with the lowest being observed for GRASS. This is the case for LM, PM and ST. In the case of SUP, however, the polyunsaturated fatty acid concentration was higher in GRASS compared to CONC, which, again, may reflect the variation in total IMF concentration for this muscle across the dietary treatments. Based on the saturated fatty acid concentration, SUP from GSL and ST from GRASS could be labelled “low in saturated fat”, i.e., saturated fatty acid concentration <1.5/100 g solid [24]. For monounsaturated fatty acids, [24] states that “a claim that a food is high in monounsaturated fat may only be made when at least 45% of the fatty acids present” are monounsaturated. Based on the proportion of monounsaturated fatty acids (Supplementary Table S2), with the exception of SUP and PM from GSS, all of the production system/treatment combinations in the present study would satisfy this criterion. For polyunsaturated fatty acids, Ref. [24] states that “a claim that a food is high in polyunsaturated fat may only be made when at least 45% of the fatty acids present” are polyunsaturated. Despite the highest (numerically) polyunsaturated fatty acid proportion being observed in all muscles due to the GRASS diet, none of them would satisfy this criterion.

### 4.3. Individual Fatty Acids and Nutritional Indices

EU Regulation [24] considers the shorter carbon chain n-3 polyunsaturated fatty acid, alpha-linoleic (LNA), and the longer carbon chain n-3 polyunsaturated fatty acids, eicosapentaenoic acid (EPA) and docosahexanoic acid (DHA), separately with respect to a nutritional claim. The concentrations required in 100 g tissue for beef to be labelled as a “source” of omega-3 fatty acids are 300 mg and 40 mg, respectively [29]. In the present study, the highest concentration of LNA was 74 mg/100 g muscle (PM from GRASS), and the highest concentration of EPA + DHA (not detected in all samples) was 29 mg/100 g muscle (PM from GRASS). The lowest concentrations of LNA and EPA + DHA were 19 and 2 mg/100 g muscle, respectively for SUP from CONC. The data indicate that the different muscles responded differently to the examined diets, e.g., there was a 3-fold increase in LNA concentration in SUP from GRASS compared to SUP from CONC, despite only a corresponding 1.8-fold increase in PM. Supplementation with a blend of linseed oil and ruminally-protected linseed increased the LNA concentration in a muscle-dependent manner (SUP and PM only), but the absolute increase was small. While the data generally demonstrate the superiority of pasture finishing, the “grass-finished” beef in the present study could not be labelled a “source” of omega-3 fatty acids as defined in [29]. Nevertheless, adherence with dietary recommendations for total fat, saturated fatty acids and polyunsaturated fatty acids would be improved if grass-fed beef replaced concentrate-fed beef in the human diet.

In recognition of the impact of a higher intramuscular fat concentration, per se, on muscle fatty acid composition [30] and to facilitate comparison with the literature, the above data were also analysed on a proportional basis. The proportion of LNA in total lipids in LM from GRASS (1.11 g/100 g) was similar to that previously reported in [31] (1.29) and in [32] (1.08) for LM from cattle that were at pasture for 158 and 200 days before slaughter in Ireland and USA, respectively. The general increase in the proportion of LNA in total lipids due to supplementation with LNA is consistent with a study by MacKintosh et al. [33].

With regard to fatty acid classes, there is a recommendation [34] on a desirable ratio of total polyunsaturated fatty acids: total saturated fatty acids on a whole-diet basis (>0.45), but this does not relate to individual foods. While there was a trend for this ratio to be higher in muscle from GRASS, as seen in the literature (e.g., [6]), this ratio was below 0.45 for all muscles in the present study. There is also a recommendation [34] on a desirable ratio of total omega-6 polyunsaturated fatty acids: total omega-3 polyunsaturated fatty acids on a whole-diet basis (<4). Other than LM, all muscles exceeded this ratio for CONC. The lower omega-6 polyunsaturated fatty acids: omega-3 polyunsaturated fatty acid ratio in all muscles from the grass-based diets—in particular, ST and LM from GRASS—may be viewed as positive in this regard.

In addition to the above ratios, indices of the “healthiness” of foods based mainly on the fatty acid composition have been proposed [15,35]. Using the atherogenic and thrombogenic indices, muscle from GSS was generally less healthy than muscle from GSL or GRASS. Based on the thrombogenic index and hypocholesterolaemic:hypercholesterolaemic indices, however, muscle from GSL was more healthy than muscle from CONC but less healthy than muscle from GRASS.

Beef contains an array of CLA isomers, of which the cis9, trans11 isomer is most prominent. Positive effects of CLA have been reported in animal models of human disease and supported—albeit not to the same extent—by human studies [36,37]. Currently, there is no reference intake for CLA. From a review of the literature, Siurana and Calsamiglia [38] concluded that with respect to human health, “an effective dose would be 0.8 g per day for the anti-carcinogenic effect, 0.6 g per day for the anti-atherosclerotic effect and 3.2 g per day for the reduction of body fat. For other effects, no specific dose has been recommended”. The concentration of CLA in muscle from CONC largely reflected the IMF concentration, and altering the production system had minor effects. This likely reflects the preferential deposition of CLA in the neutral lipid fraction of muscle [30]. When expressed on a proportional basis, increases were observed due to linseed supplementation and pasture feeding, supporting previous observations [31,39]. The CLA proportion in LM from GRASS (0.49) was similar to that previously reported [31,32] (0.71 and 0.69 g/100 g, respectively) but was two-fold higher in PM from GRASS. If the contribution to the CLA concentration due to the desaturation of C18:1trans11 (also highest in PM from GRASS) to CLAcis9, trans11 in human tissue (20–25%, [40]), is considered, an average PM steak (200 g) from GRASS could supply approximately 0.2 g CLA. This would make a substantial contribution to the effective dose reported in [38].

There is considerable interest in the effect on human health of the consumption of trans fatty acids [41]. Research on ruminally-derived trans fatty acids, among which C18:1trans11 predominates, suggests either a positive or neutral effect on human health compared to the detrimental effects of industrially derived trans fatty acids, which have a higher proportion of C18:1trans10 and a more diverse profile [42]. Generally, grass feeding or supplementation with linseed oil increases the proportion of C18:1trans11 in total lipids, as seen in the present study. There is no reference intake value for C18:1trans11 currently.

### 4.4. Discrimination According to Dietary Treatment or Muscle Type

One goal of testing the potential of the fatty acid profile to authenticate the dietary history was to confirm what has previously been reported, i.e., that grass-fed beef and concentrate-fed beef can be clearly separated [6,43], which was the case in the present study. It is recognised that in this study, the diet of the animal was confounded with the age of the animal; therefore, if the data were classified based on age, a similar separation would have been achieved. This highlights a challenge with this particular approach, and for practical application, a more sophisticated model incorporating the age of the animal would seem to be required. In this study, the grass silage-fed animals were a similar age at slaughter. The nutritional enhancement was sufficient to allow for discrimination based on the fatty acid profile of beef. A similar observation was made for beef from cattle fed grass only and grass supplemented with linseed oil [6]. This indicates the potential to develop a tool to authenticate beef that is nutritionally enhanced by supplementation with LNA other than by grass feeding. The primary goal in the discrimination analysis, however, was in determining if the fatty profile could be used to discriminate between muscles of different commercial value. Such a tool would allow for assurance to the consumer that a more expensive muscle such as PM is genuine and prevent fraudulent substitution of PM with a cheaper muscle. This was moderately successful with respect to the separation of PM, ST and SUP. However, the classification results, combined with the CV-LOO predictions, indicate that discrimination between the more valuable LM from the lower value ST is more challenging and that other approaches are required to achieve this.

## 5. Conclusions

This study confirmed that within an early-maturing breed heifer production system, beef that could be labelled as “grass-fed”, i.e., only ever consuming grass or grass-based feed ingredients, had a higher concentration of nutritionally beneficial components than beef that could be labelled as “grass-based”. The array of muscles examined in this study, expanding on previous studies, and the information generated on the interactions between production system and muscle type will assist in the labelling and marketing of individual muscles from grass-fed” beef. This study confirmed the utility of the fatty acid profile as a tool to authenticate the dietary background of beef. This study also demonstrated that the fatty acid profile can be used to discriminate between some muscles. However, discriminating between all the examined muscles proved more challenging than discriminating between dietary backgrounds, and additional analytical approaches are required to improve this.

## Figures and Tables

**Table 1:** Concentrate formulation and feed composition.

	Standard Concentrate		Linseed Concentrate		Grass		Medium Silage		High Silage		Finishing Silage	
	Mean	SD	Mean	SD	Mean	SD	Mean	SD	Mean	SD	Mean	SD
Ingredient (g/kg)												
Rolled barley	862		813									
Linseeds			46									
Soyabean meal	60		57									
Linseed oil			14									
Cane molasses	50		47									
Mineral/Vitamin Mix	28		27									
Chemical composition												
Dry matter (DM, g/kg)	793	(7.3)	827	(11.4)	208	(20.3)	211	(31.2)	289	(69.8)	251	(15.2)
Ash ^1^	58	(5.8)	77	(10.5)	86	(9.1)	77	(7.0)	101	(2.8)	80	(6.0)
Crude protein ^1^	125	(8.0)	126	(12.5)	203	(29.4)	162	(6.1)	136	(1.8)	166	(5.3)
Oil B ^1,2^	24	(2.0)	59	(16.6)	40	(8.9)	36	(2.0)	42	(8.4)	48	(2.5)
DM digestibility (g/kg)	-				800	(27.1)	699	(24.2)	767	(19.0)	778	(16.2)
NCGD (g/kg) ^3^	839	(20.6)	791	(20.5)	-	-	-	-	-	-	-	-
NDF ^1,4^	193	(22.4)	203	(11.7)	428	(21.4)	515	(29.2)	426	(8.4)	425	(45.3)
β-carotene (mg/kg) ^1^	ND		ND		26	(25.8)	7	(2.5)	8	(2.0)	35	(9.5)
Vitamin E (mg/kg) ^1^	13	(13.8)	14	(4.7)	12	(10.9)	27	(14.8)	15	(2.1)	39	(5.0)
Fatty acids (g/kg fatty acids)												
C12:0	6.6	(6.22)	1.7	(1.60)	1.7	(0.36)	2.6	(0.43)	2.4	(0.28)	1.8	(0.18)
C14:0	6.7	(2.09)	1.9	(0.96)	4.4	(0.93)	9.0	(2.61)	6.2	(0.18)	5.7	(0.37)
C16:0	265.8	(20.65)	106.3	(14.61)	134.9	(18.23)	144.7	(11.25)	138.7	(5.15)	126.0	(3.50)
C16:1	2.7	(0.33)	1.1	(0.52)	1.9	(1.00)	4.1	(1.51)	3.8	(0.62)	2.4	(0.19)
C18:0	18.1	(2.98)	33.4	(1.39)	15.1	(2.13)	15.7	(1.37)	13.8	(0.66)	12.2	(0.61)
C18:1	148.8	(13.93)	182.1	(8.14)	15.3	(3.38)	20.5	(3.24)	19.0	(2.48)	14.0	(0.69)
C18:2	430.0	(43.79)	241.8	(17.11)	89.0	(7.06)	111.6	(4.84)	109.6	(3.60)	110.8	(0.95)
C18:3	31.0	(5.70)	374.6	(38.68)	457.1	(50.63)	348.1	(44.29)	413.9	(16.65)	442.7	(19.87)
C20:0	2.5	(0.55)	1.9	(0.16)	4.6	(1.07)	5.8	(0.58)	4.6	(0.29)	3.8	(0.09)
C20:1	6.9	(0.70)	0.6	(1.11)	0.6	(0.10)	1.0	(0.23)	0.8	(0.33)	0.5	(0.07)
C20:2	1.1	(0.33)	0.4	(0.20)	3.8	(1.92)	1.2	(0.57)	1.0	(0.31)	0.7	(0.11)
C22:0	2.7	(0.48)	2.1	(0.55)	9.4	(1.92)	11.0	(0.89)	8.5	(0.77)	8.9	(0.32)

^1^ g/kg DM. ^2^ Acid hydrolysis, ether extract. ^3^ Neutral cellulase gammanase digestibility. ^4^ Neutral detergent fibre.

**Table 2:** Growth and carcass characteristics of heifers finished on concentrates ad libitum (CONC), grass silage and standard concentrate (GSS), grass silage and linseed concentrate (GSL) or grazed grass (Grass) ^1^.

	Treatment					
	CONC	GSS	GSL	Grass	Sed ^2^	Significance
Weight (kg)						
Initial	296	297	297	296	2.3	NS
Final	479 ^a^	516 ^b^	530 ^b^	510 ^b^	12.7	**
Duration (days)	188	293	294	336	-	-
Age at slaughter (days)	465 ^a^	567 ^b^	581 ^b^	630 ^c^	11.9	***
Overall growth (g/day)	978 ^a^	747 ^b^	790 ^b^	636 ^c^	47.6	***
Carcass weight (kg)	258	265	269	267	6.4	NS
Conformation ^3^	7.1	6.5	7.2	6.9	0.45	NS
Fat classification ^4^	10.3	9.7	10.0	10.6	0.512	NS

^1^ In this and subsequent tables, n = 3 groups of 5 animals per treatment. ^2^ sed = standard error of the difference between means. Means within a row with different letters differ significantly at *p* < 0.05. ^3^ 15-point scale; 1 = poorest. ^4^ 15-point scale; 1 = leanest. NS = not significant; ** = *p* < 0.01; *** = *p* < 0.001.

**Table 8:** Classification results for discrimination models for diet and muscle type ^1^.

	Diet ^2^ Discrimination					Muscle ^3^ Discrimination				
		Predictions					Predictions			
		CONC	GSS	GSL	Grass		SUP	ST	LM	PM
Training	CONC (n = 42)	42.0	0.0	0.0	0.0	SUP (n = 43)	37.0	1.7	1.0	3.3
	GSS (n = 41)	0.0	39.3	1.7	0.0	ST (n = 42)	2.3	31.7	6.3	1.7
	GSL (n = 42)	0.0	0.0	42.0	0.3	LM (n = 43)	3.3	2.7	34.7	2.3
	Grass (n = 45)	0.0	0.3	3.3	41.3	PM (n = 43)	2.7	0.7	0.7	39.0
	Sensitivity (%)	100.0	95.9	100.0	91.9	Sensitivity (%)	86.0	75.4	80.6	90.7
	Specificity (%)	100.0	99.7	96.1	99.7	Specificity (%)	93.5	96.1	93.8	94.3
	Accuracy (%)	96.9				Accuracy (%)	83.2			
CV-LOO	CONC (n = 42)	42.0	0.0	0	0	SUP (n = 43)	34.0	2.0	2.0	5.0
	GSS (n = 41)	0.0	36.3	3.3	1.3	ST (n = 42)	4.0	26.7	9.0	2.3
	GSL (n = 42)	0	1.3	38.0	2.7	LM (n = 43)	4.7	4.7	31.0	2.7
	Grass (n = 45)	0	0.7	5.0	39.3	PM (n = 43)	3.0	1.0	1.7	37.3
	Sensitivity (%)	100.0	88.6	90.5	87.4	Sensitivity (%)	79.1	63.5	72.1	86.8
	Specificity (%)	100.0	98.4	93.5	96.8	Specificity (%)	90.9	94.1	90.1	92.2
	Accuracy (%)	91.6				Accuracy (%)	75.4			
Test	CONC (n = 14)	14.0	0.0	0.0	0.0	SUP (n = 14)	11.0	0.3	0.3	2.3
	GSS (n = 14)	0.0	11.7	2.0	0.3	ST (n = 14)	0.7	9.0	2.7	1.7
	GSL (n = 14)	0.0	0.7	12.0	1.3	LM (n = 14)	0.3	1.7	11.7	0.3
	Grass (n = 15)	0.0	0.3	2.7	12.0	PM (n = 14)	1.7	0.3	0.3	11.7
	Sensitivity (%)	100.0	83.3	85.7	80.0	Sensitivity (%)	78.6	64.3	83.3	79.8
	Specificity (%)	100.0	97.7	89.1	96.0	Specificity (%)	93.7	94.4	92.1	89.7
	Accuracy (%)	87.1				Accuracy (%)	77.4			

^1^ Results are the average of 3 repeats resulting from random splitting of the data into training and test set 3 times (ratio = 0.75). ^2^ Concentrates ad libitum (CONC), grass silage and standard concentrate (GSS), grass silage and linseed concentrate (GSL) or grazed grass (Grass). ^3^ SUP = supraspinatus (chuck tender); ST = semitendinosus (eye of the round); LM = longissimus muscle (striploin); PM = psoas major (fillet).

## Data Availability

The original contributions presented in this study are included in the article/Supplementary Materials. Further inquiries can be directed to the corresponding author.

## Supplementary Materials

The following supporting information can be downloaded at https://www.mdpi.com/article/10.3390/foods14050747/s1: Table S1. Total concentrations and proportions (g/100 g) of fatty acids in uncooked or sous vide cooked longissimus muscle from heifers finished on concentrates ad libitum (CONC) or grazed grass (Grass). Table S2. Proportions of nutritionally relevant fatty acids in muscles from heifers finished on concentrates ad libitum (CONC), grass silage and standard concentrate (GSS), grass silage and linseed concentrate (GSL) or grazed grass (Grass).
