# Supplementary material for: The Concentrations of Fatty Acids, Cholesterol and Vitamin E in Cooked Longissimus, Semitendinosus, Psoas Major and Supraspinatus Muscles from Cattle Offered Grass Only, Concentrates Ad Libitum or Grass Silage Supplemented with Concentrates
Source: Foods. 2025 Feb 22;14(5):747. doi: 10.3390/foods14050747 (PMC11899229; doi:10.3390/foods14050747)
Supplement: Supplementary file 1 [file foods-14-00747-s001.zip › foods-3412729-supplementary.pdf]

Supplementary Table S1. Total concentration and proportion (g/100g) of fatty acids[1] in uncooked or sous-vide cooked longissimus muscle from heifers finished on concentrates *ad libitum* (CONC) or grazed grass (Grass)

	Diet					Significance		
	CONC		Grass					
	Cooked	Uncooked	Cooked	Uncooked	Sed[2]	Diet	Cooking	Diet*Cooking
Total Fatty Acid (mg/g)	75.2	48.9	53.8	36.1	4.43	***	***	NS
C14:0	2.51	2.36	2.46	2.42	0.11	NS	*	NS
C15:0 i	0.11	0.10	0.21	0.20	0.01	***	*	NS
C15:0 ai	0.14	0.12	0.23	0.23	0.01	***	NS	NS
C14:1	0.59	0.57	0.56	0.56	0.04	NS	NS	NS
C15:0	0.40	0.37	0.55	0.53	0.03	***	**	NS
C15:1	0.05	0.05	0.10	0.11	0.01	***	NS	NS
C16:0 iso	0.08	0.09	0.01	0.01	0.01	***	NS	NS
C16:0	22.79	21.93	20.72	20.28	0.45	***	***	NS
C17:0i+C16:1t9	0.35	0.34	0.57	0.57	0.02	***	NS	NS
C16:1t10 to 12	0.24	0.25	0.33	0.32	0.01	***	NS	NS
C16:1c9+c17:0ai	3.53	3.47	3.21	3.21	0.14	0.053	NS	NS
C16:1 c13	0.16	0.16	0.12	0.13	0.01	*	NS	NS
C17:0	1.10	1.04	1.13	1.10	0.04	NS	***	NS
C17:1 c11	0.95	0.95	0.83	0.85	0.05	*	NS	NS
C18:0	13.12	12.80	14.81	14.61	0.33	***	**	NS
C18:1 t6-8	0.11	0.09	0.14	0.13	0.01	***	*	NS
C18:1 t9	0.19	0.19	0.22	0.21	0.01	*	NS	NS
C18:1 t10	0.31	0.24	0.15	0.16	0.04	**	NS	*
C18:1 t11	0.94	0.90	2.71	2.60	0.23	***	**	NS
C18:1t12+C18:1t13	0.16	0.14	0.20	0.19	0.01	***	*	NS
C18:1 c9	37.89	37.37	32.14	31.82	0.64	***	*	NS
C18:1 c11	1.79	1.81	1.33	1.34	0.06	***	NS	NS
C18:1 c12	0.11	0.11	0.08	0.09	0.01	***	NS	NS
C18:1 c13	0.45	0.44	0.31	0.32	0.02	***	NS	NS
C18:1 t16	0.16	0.15	0.27	0.26	0.01	***	**	NS
C18:1c15; C18:2 10, 14; C19:0	0.17	0.17	0.23	0.23	0.01	***	NS	NS

Supplementary Table S1 (cont'd). Total concentration and proportion (g/100g) of fatty acids[1] in uncooked or sous-vide cooked longissimus muscle from heifers finished on concentrates *ad libitum* (CONC) or grazed grass (Grass)

	Diet					Significance		
	CONC		Grass					
	Cooked	Uncooked	Cooked	Uncooked	Sed[2]	Diet	Cooking	Diet*Cooking
C18:2 10,13;C18:2 11,14	0.17	0.16	0.26	0.26	0.02	***	NS	NS
C18:2 t9,c15	0.09	0.08	0.06	0.06	0.00	***	NS	NS
C18:1 c16	0.16	0.14	0.16	0.14	0.01	***	0.053	NS
C18:2 t10,c15	0.14	0.13	0.42	0.40	0.03	***	*	NS
C18:2 c9,12	3.36	3.79	2.69	2.89	0.22	**	***	NS
C18:2 t12,c15	0.17	0.17	0.07	0.07	0.01	***	NS	NS
C20:0	0.07	0.06	0.09	0.09	0.01	**	NS	NS
C18:3 (n6)	0.09	0.09	0.03	0.03	0.01	NS	NS	NS
C20:1 t15	0.09	0.09	0.06	0.07	0.01	*	NS	NS
C20:1 c11	0.12	0.12	0.11	0.10	0.02	NS	NS	NS
C18:3 c9,12,15 (n3)	0.50	0.54	1.65	1.75	0.08	***	*	NS
C18:2 c9,t11(CLA)	0.41	0.42	0.72	0.74	0.06	***	NS	NS
C21:0	0.02	0.02	0.03	0.03	0.01	NS	NS	NS
C18:2 t10,c12(CLA)	0.04	0.04	0.13	0.12	0.01	***	NS	NS
C20:2	0.04	0.04	0.05	0.04	0.01	NS	NS	NS
C22:0	0.03	0.02	0.08	0.07	0.03	*	NS	NS
C20:3n6	0.36	0.43	0.18	0.21	0.04	***	**	NS
C20:4 c5,8,11,14	0.96[a]	1.23[b]	0.85[a]	0.95[a]	0.09	*	***	*
EPA	0.20	0.25	0.71	0.79	0.05	***	**	NS
C22:5	0.07	0.09	0.14	0.15	0.01	***	*	NS
DHA	0.03	0.03	0.04	0.05	0.01	***	NS	NS
SFA	40.83	39.41	41.04	40.31	0.58	NS	***	NS
MUFA	47.99	47.23	43.08	42.66	0.60	***	**	NS
PUFA	6.57	7.43	7.97	8.50	0.44	*	***	NS

[1]CLA = conjugated linoleic acid; EPA = eicosapentaenoic acid; DHA = docosahexanoic acid; SFA = total saturated fatty acids; MUFA = total monosaturated fatty acids; PUFA = total polyunsaturated fatty acids; [2]Sed = standard error of the difference. Means within a row with different letters differ significantly at $P < 0.05$.

Supplementary Table S2. Proportions of nutritionally relevant fatty acids[1] in muscles[2] from heifers finished on concentrates *ad libitum* (CONC), grass silage and standard concentrate (GSS), grass silage and linseed concentrate (GSL) or grazed grass (Grass)

		Treatment					
	Muscle	CONC	GSS	GSL	Grass	Sed[3]	Significance
%SFA	SUP	41.38^{b}	43.07^{c}	38.67^{a}	38.22^{a}	0.690	***
	ST	39.49^{a}	45.21^{d}	43.94^{c}	42.44^{b}	0.613	***
	LM	40.98^{a}	44.90^{c}	42.19^{b}	41.40^{ab}	0.542	***
	PM	43.97^{a}	46.70^{b}	44.52^{a}	44.57^{a}	0.537	***
%MUFA	SUP	49.67^{c}	44.63^{a}	46.09^{ab}	46.26^{b}	0.670	***
	ST	52.34^{c}	46.72^{a}	46.91^{ab}	48.17^{b}	0.710	***
	LM	49.24^{a}	48.28^{a}	51.16^{b}	50.93^{b}	0.623	***
	PM	47.81^{c}	43.89^{a}	46.82^{c}	45.54^{b}	0.584	***
%PUFA	SUP	6.48^{a}	6.85^{a}	7.54^{b}	7.88^{b}	0.320	***
	ST	5.91^{a}	6.06^{a}	6.06^{a}	7.22^{b}	0.455	*
	LM	5.57^{b}	4.69^{a}	5.46^{b}	5.87^{b}	0.354	*
	PM	4.79^{a}	5.99^{b}	5.82^{b}	6.66^{c}	0.357	***
%LNA	SUP	0.41^{a}	0.86^{b}	1.21^{c}	1.46^{d}	0.052	***
	ST	0.43^{a}	0.98^{b}	1.21^{c}	1.30^{c}	0.089	***
	LM	0.36^{a}	0.75^{b}	1.00^{c}	1.11^{c}	0.059	***
	PM	0.48^{a}	0.99^{b}	1.20^{c}	1.50^{d}	0.071	***
%LA	SUP	3.37^{c}	2.36^{a}	2.82^{b}	2.50^{ab}	0.164	***
	ST	2.54	2.56	2.20	2.09	0.236	NS
	LM	2.29^{c}	1.83^{ab}	2.02^{bc}	1.69^{a}	0.134	***
	PM	2.67	2.52	2.47	2.42	0.171	NS
%CLA	SUP	0.28^{a}	0.53^{b}	0.56^{b}	0.87^{c}	0.066	***
	ST	0.38^{a}	0.36^{a}	0.46^{a}	0.80^{b}	0.109	***
	LM	0.27^{a}	0.53^{b}	0.77^{c}	0.49^{b}	0.098	***
	PM	0.46^{a}	0.47^{a}	0.73^{b}	0.95^{c}	0.070	***
%VA	SUP	0.90^{a}	1.72^{b}	1.58^{b}	2.60^{c}	0.196	***
	ST	0.75^{a}	1.85^{b}	2.01^{b}	3.08^{c}	0.219	***
	LM	0.64^{a}	1.57^{b}	1.74^{b}	2.85^{c}	0.215	***
	PM	1.20^{a}	2.04^{b}	2.23^{b}	3.54^{c}	0.217	***
%EPA	SUP	0.04^{a}	0.33^{b}	0.60^{C}	0.69^{c}	0.046	***
	ST	0.28^{a}	0.56^{bc}	0.54^{b}	0.80^{c}	0.115	**
	LM	0.20^{ab}	0.07^{b}	0.18a^{b}	0.33^{b}	0.090	*
	PM	0.02^{a}	0.22^{b}	0.38^{c}	0.61^{d}	0.070	***

[1]SFA = total saturated fatty acids; MUFA = total monounsaturated fatty acids; PUFA = total polyunsaturated fatty acids; LNA = linolenic acid; LA = linoleic acid; CLA = conjugated linoleic acid; VA = vaccenic acid; EPA = eicosapentaenoic acid.

[2]SUP = supraspinatus (chuck tender); ST = semitendinosus (eye of the round); LM = longissimus muscle (striploin); PM = psoas major (fillet).

[3]sed = standard error of the difference. Means within a row with different letters differ significantly at $P < 0.05$.
